# Supplementary material for: Deciphering the Genomic Landscape and Pharmacological Profile of Uncommon Entities of Adult Rhabdomyosarcomas
Source: Int J Mol Sci. 2021 Oct 26;22(21):11564. doi: 10.3390/ijms222111564 (PMC8584142; doi:10.3390/ijms222111564)
Supplement: Supplementary file 1 [file ijms-22-11564-s001.zip › ijms-1414269-supplementary.pdf]

Supplemetary Materials

Table S1. Primers and probes used for Real-time PCR analysis.

**SYBR™ Green Assays**		
Gene	Forward primer (5’-3’)	Reverse primer (5’-3’)
TP53I3	**AAT-GCT-TTC-ACG-GAG-CAA-ATT-C**	**TTC-GGT-CAC-TGG-GTA-TCT**
LAPTM4A	**GCC-TGT-GTT-CTT-TTT-CCC-GTC-T**	**GGA-ATC-AGC-CAA-CCC-ACT-TGA**
RAB22A	**CAC-CTT-ACC-GTC-TCT-GTG-ATT-G**	**GTC-TGT-GGC-CAT-TCT-TTA**
S100P	**TGC-AGA-GTG-GAA-AAG-ACA-AGG-AT**	**CCA-CCT-GGG-CAT-CTC-CAT-T**
CD109	**CCT-GTG-ACC-TTT-GCA-GTG-ATG-T**	**GAG-TGA-TGG-GAG-CCT-GAA**
LAPTM4B	**GTT-ACC-AGC-AAT-GAC-ACT-ACG-G**	**CTC-CTT-GGC-AGC-ACC-ATT**
GAPDH	**ACC-ACA-GTC-CAT-GCC-ATC**	**TCC-ACC-ACC-CTG-TTG-CTG-TA**
**TaqMan®Assays**		
Gene	Assay identification number	
VIM	Hs_00185584_m1	
CDH1	Hs_01023894_m1	
TGFb	Hs_00998133_m1	
SNAIL1	Hs_00195591_m1	
MMP2	Hs_01548727_m1	
MMP9	Hs_00234579_m1	
SLUG	Hs_00161904_m1	
HPRT	Hs_99999909_m1	

Table S2. Target gene analyzed for microsatellite instability (MSI) status.

**Marker**	**Gene**	**NCBI Ref. Seq. (GRCh 38.p12)**	**Choromosom**
BAT25	*cKIT*	NC_000004.12	4(4q12)
BAT26	*MSH2*	NC_000002.12	2(2p21-p16.3)
NR21	*SLC7A8*	NC_000014.9	4(14q11.2)
NR22	*STT3A*	NC_000011.10	11(11q24.2)
NR24	*ZNF2*	NC_000002.12	2(2q11.1)
NR27	*BIRC3*	NC_000011.10	11(11q22.2)
CAT25	*CASP2*	NC_000007.14	7(7q34)
MONO27	*MAP4K3*	NC_000002.12	2(2p22.1)

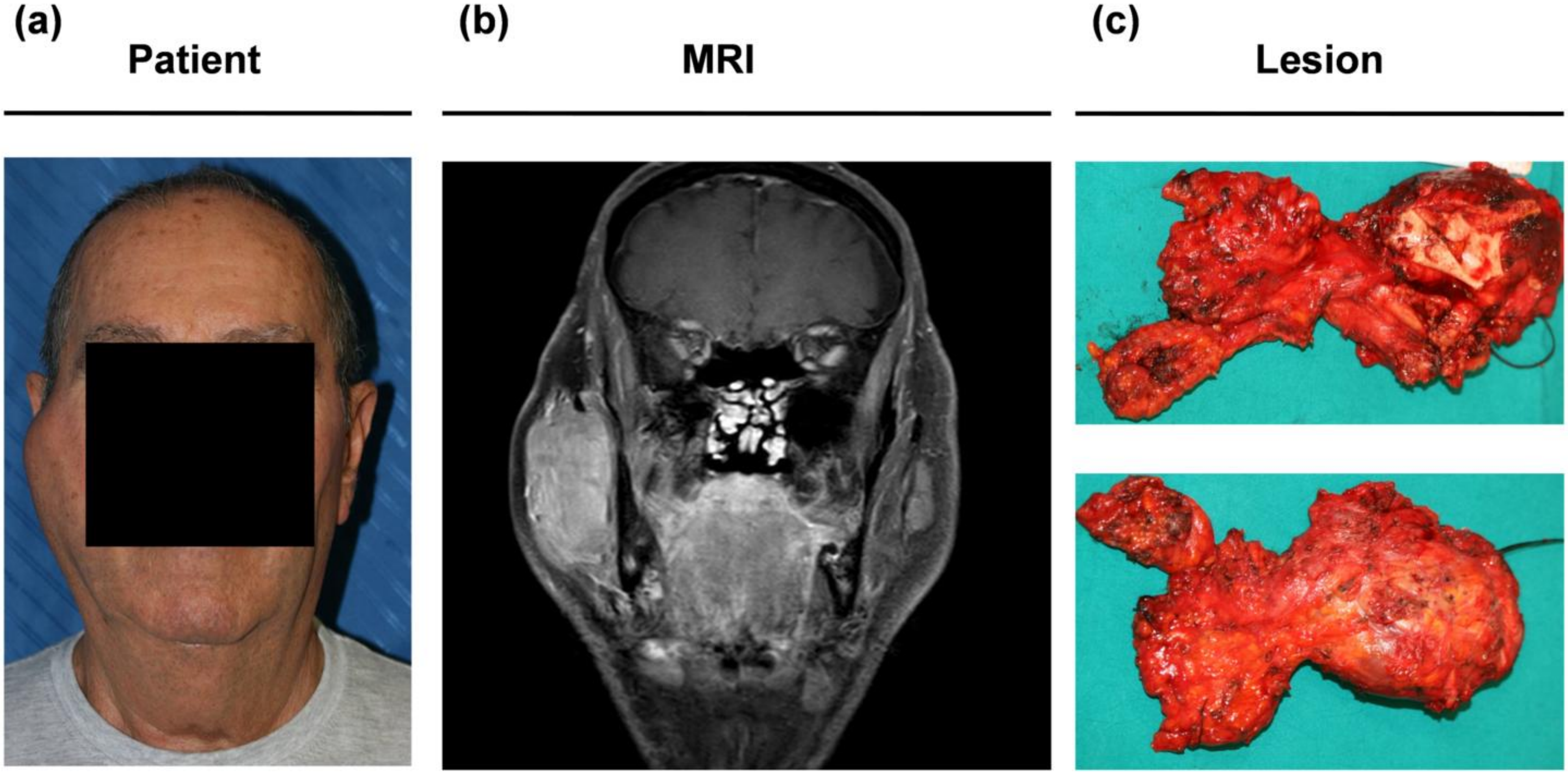

Supplementary Figure S1. (a) Frontal view of patient face. **(b)** Coronal MRI. **(c)** Lesion surgically excised.

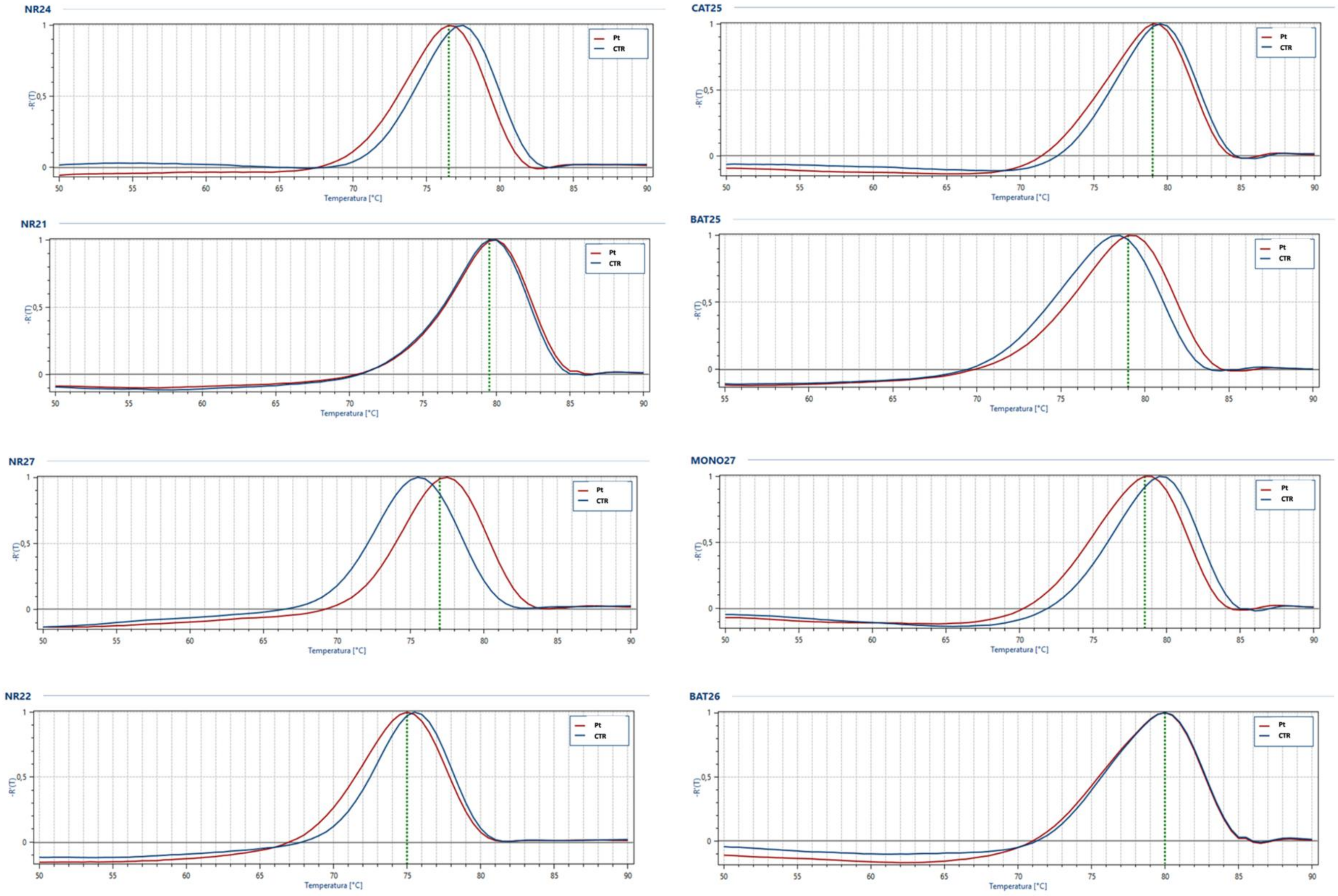

Supplementary Figure S2. Representative melt curves of BAT-25, BAT-26, NR-21, NR-22, NR-24, NR-27, CAT-25, MONO-27 (see complete list of gene in Supplementary Table S1) in the RMS patient tumor sample (Pt, red line) compared to the stable positive control (CTR, blue line).

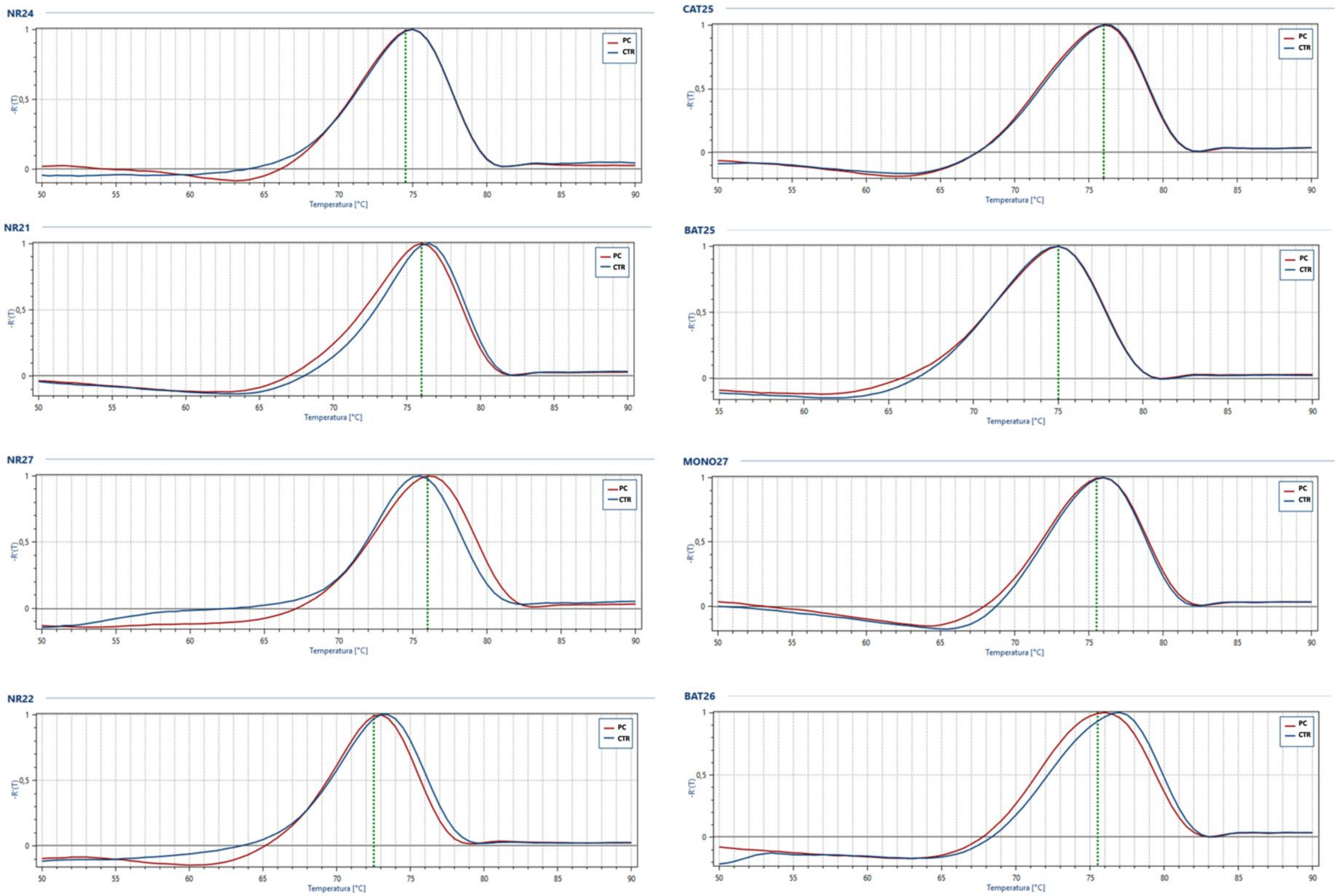

Supplementary Figure S3. Representative melt curves of BAT-25, BAT-26, NR-21, NR-22, NR-24, NR-27, CAT-25, MONO-27 (see complete list of gene in Supplementary Table S1) in the RMS primary culture (PC, red line) compared to the stable positive control (CTR, blue line).

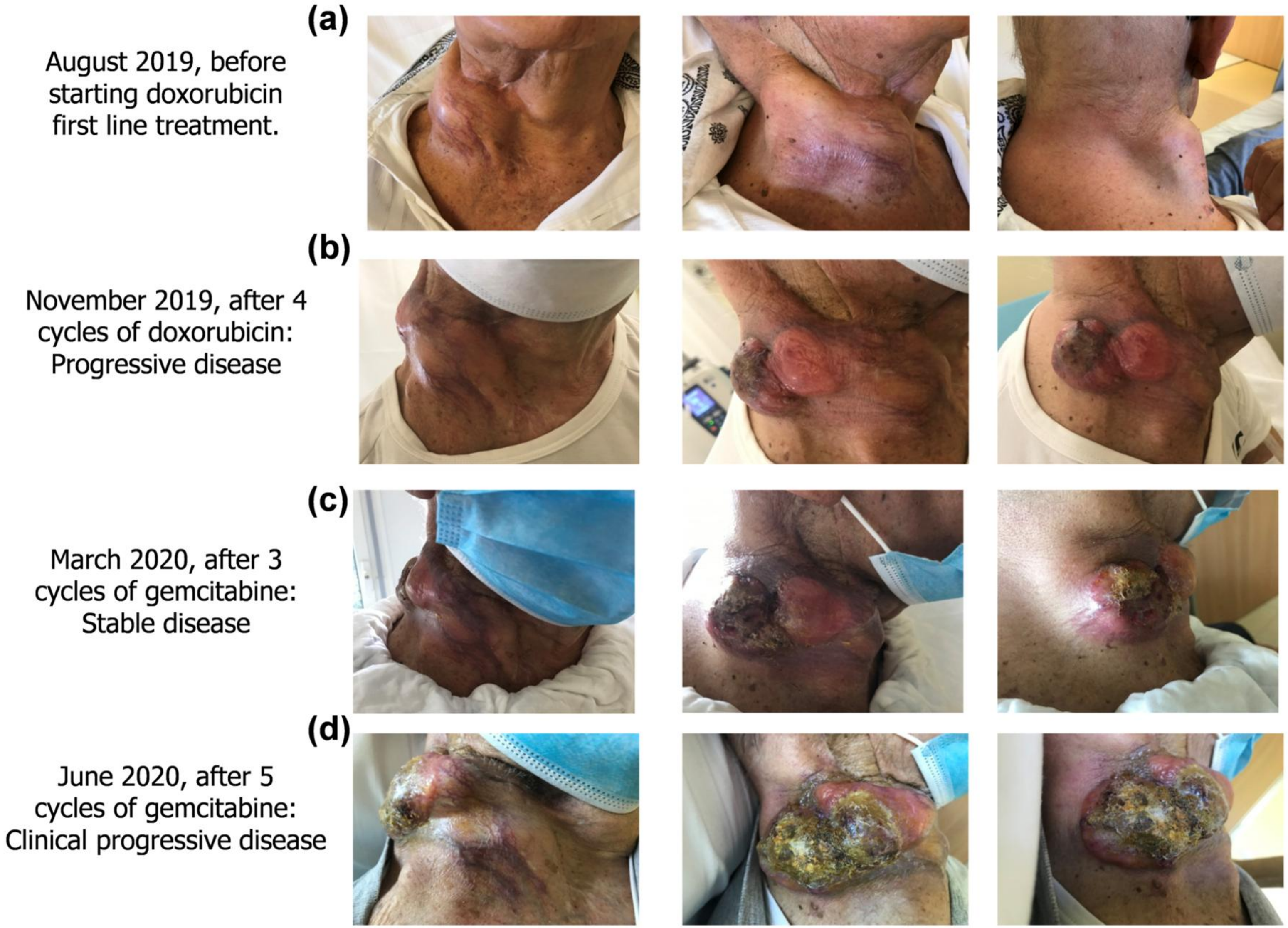

Supplementary Figure S4. Clinical and radiological evaluation of disease showing: **(a)** clinical and radiological presentation of local relapse three months after adjuvant radiotherapy. **(b)** clinical and radiological progression of disease after four cycles of doxorubicin.**(c)** clinical and radiological stable disease after 3 cycles of gemcitabine. **(d)** clinical progression of disease that led to treatment discontinuation.

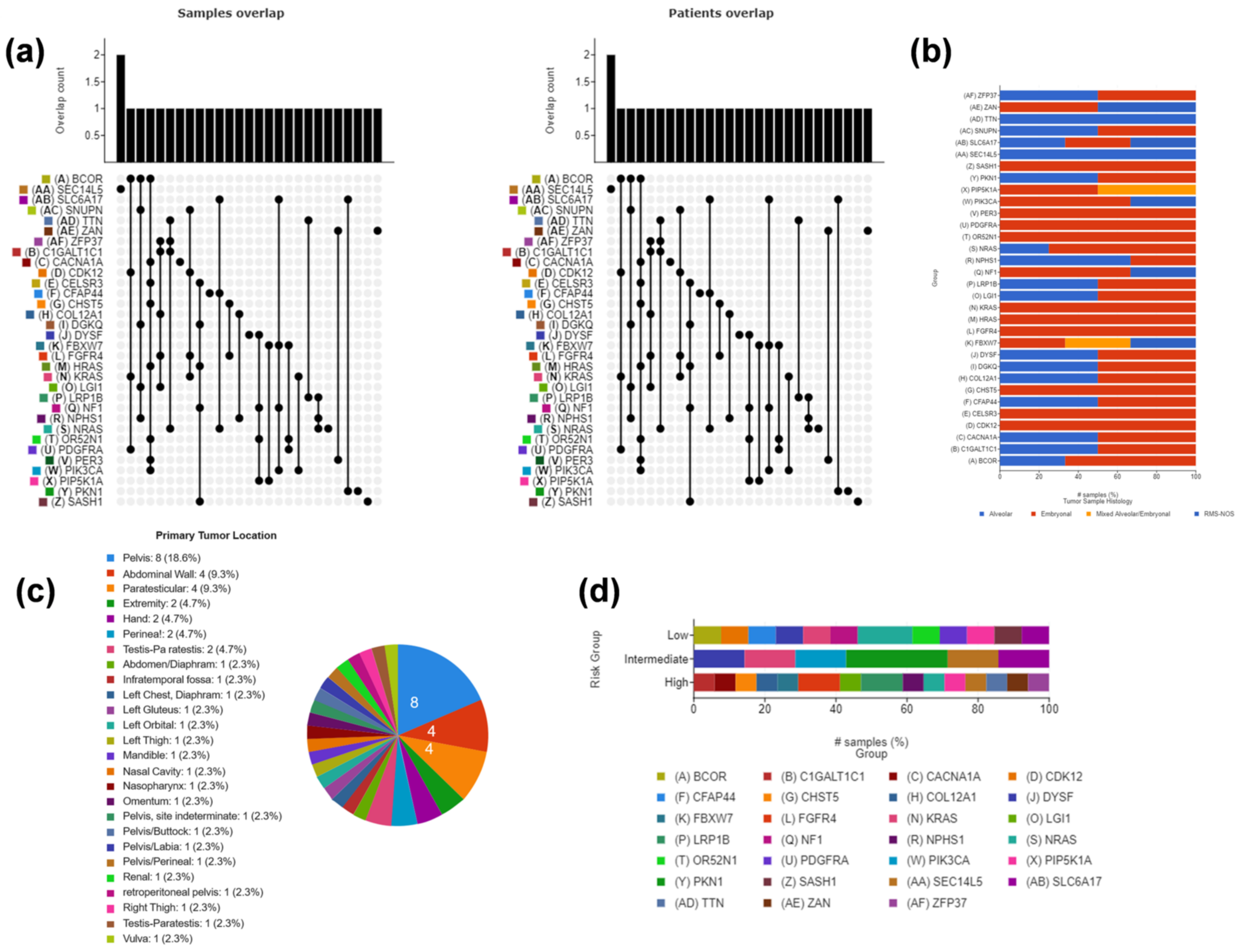

Supplementary Figure S5. (a) In silico analysis among 43 RMS patient showing the overlapping among tumor and patient samples of the most mutated genes in case study. **(b)** Correlation between the most mutated genes and RMS tumor histology. **(c)** Primary tumor location in the in silico case study. **(d)** Correlation between the most mutated genes and RMS risk group.
